# A chicken microRNA expressing LaSota targets Newcastle disease virus phosphoprotein and impacts interferon expression

**DOI:** 10.1016/j.psj.2026.107893

**Published:** 2026-10-05

**Authors:** Abhijeet Bakre, Timothy R. Satterlee, Megan C. Mears

**Affiliations:** a Exotic and Emerging Avian Viral Disease Research Unit, Southeast Poultry Research Laboratories, US National Poultry Research Center, 934 College Station Road, Athens, GA, 30605, United States; b Core Sequencing Lab, US National Poultry Research Center, 950 College Station Road, Athens, GA, 30605, United States

**Keywords:** Newcastle disease, Newcastle disease virus, microRNAs, Phosphoprotein

## Abstract

Newcastle disease (ND), a major cause of economic loss in the poultry industry worldwide, is caused by outbreaks of virulent avian paramyxovirus type 1(APMV-1) virus. We previously demonstrated that chicken miRNA-27b-5p (gga-miR-27b-5p) can regulate the APMV-1 phosphoprotein (P) and alter viral replication. In this study, the LaSota vaccine strain of APMV-1 was used as a backbone to express the chicken pre-miRNA-27b and expected to increase intra-cellular levels of both gga-miR-27b-5p and gga-miR-27b-3p. This novel recombinant virus, rLS-miR-27b, was rescued, propagated in embryonating chicken eggs (ECEs) and did not exhibit toxicity in ECEs. Expression of both gga-miR-27b-5p and gga-miR-27b-3p was increased significantly relative to controls. An inverse correlation was observed between increased expression of gga-miR-27b-5p and reduced APMV-1 P transcripts. Additionally, increased expression of interferons (alpha, beta and lambda) and interferon stimulated gene MX1 was observed. Suppression of P did not alter the P:V ratio in rLS-27b virus infected cells. These data support the observation that miR-27b-5p expression alters viral transcriptional kinetics in cell culture without affecting overall progeny virus yield in ECEs and could be utilized to improve current APMV-1 vaccines.

## Introduction

Newcastle disease (ND) is a reportable disease of wild birds and poultry caused by virulent strains of avian paramyxovirus type-1 (APMV-1) (WOAH, 2026). The ∼15.2 kb long negative-sense single stranded RNA (-ssRNA) APMV-1 genome encodes six genes (nucleocapsid (N), phosphoprotein (P), matrix (M), fusion (F), hemagglutinin-neuraminidase (HN), and polymerase (L)) (Dimitrov et al., 2019) and current F based phylogenetics divide all APMV-1 viruses into class I or class II viruses (Dimitrov et al., 2019). The class II viruses presently contain 21 genotypes of which genotypes V and VII are often associated with ND outbreaks (Dimitrov et al., 2019) globally. Low virulence strains of APMV-1 are globally endemic in some species of waterfowl that serve as a reservoir for the virus, produce subclinical to mild disease, and are not reportable. Despite this genetic diversity, all APMV-1 s belong to a single serotype.

ND presents suddenly (24-48 hours after infection) with high mortality often reaching 50-100 % in naïve flocks. Typical signs of ND can be respiratory (sneezing, coughing, and/or respiratory distress), digestive (greenish watery diarrhea) or neurological (drooping wings, torticollis, and/or convulsions) (WOAH, 2026, Dimitrov, 2024). ND outbreaks cause serious economic damage and lead to trade embargoes and (Dimitrov, 2024). Currently, an ongoing ND outbreak is causing extensive economic losses in Poland and Germany, while Chile, Brazil, and China have had recent ND outbreaks contributing to economic losses (WOAH, 2026). WOAH standards mandate that ND outbreaks be immediately reported; in the U.S. the infected flocks must be depopulated (often within 24 h), all movement of poultry, poultry products and associated materials is strictly prohibited, and a surveillance zone must be established with a 10 km (6.2 mile) radius to monitor virus transmission (USDA, 2014). This biosecurity and depopulation based approach can prevent further spread of APMV-1 viruses which can remain viable in the environment. However, both biosecurity and depopulation are expensive options especially for poultry farmers in low resource countries.

Current ND vaccination programs use Live attenuated vaccines (LAVs), inactivated vaccines or vaccines that use the herpes virus of turkey (HVT) backbone (Bello et al., 2018). These strategies that rely on triggering a strong humoral and or cellular response to surface F and HN antigens of APMV-1, reduce morbidity and mortality however fail to reduce viral shedding or require higher boosts following repeated infection (Dimitrov et al., 2017). Considering the environmental stability of APMV-1, vaccines that block or reduce transmission either by reducing viral replication or egress can help in ND containment.

Previously, RNA interference (RNAi) mediated knock down of APMV-1 NP (Yin et al., 2010, Yue et al., 2009) but not P (Yin et al., 2010; Yue et al., 2009; Yang et al., 2016; Yue et al., 2008) demonstrated reduction in viral replication without cellular toxicity (Hutcheson et al., 2015). However, since RNA viruses mutate at higher rates, targeting single viral genes may lead to emergence of escapee viruses reducing the efficacy of RNAi. Targeting a combination of viral and host genes that are essential for viral survival can significantly reduce this escape. Prior studies have validated a class of non-coding RNAs, known as microRNAs (miRs), as important regulators of viral replication both by directly targeting viral genes or by regulating host genes and pathways critical for viral replication (Chen et al., 2020; Chowdhury et al., 2024; Teng et al., 2023; Yao and Nair, 2014; Zhuang et al., 2017). MiRs are highly conserved, 18-25 nt long non-coding RNAs and a single miR can down-regulate expression of hundreds of genes post-transcriptionally (Duchaine and Fabian, 2019). Canonical miR biosynthesis produces hairpin structured pre-miR transcripts from RNA Pol II transcription; each pre-miR transcript encodes two forms of the miR, the more abundant form designated as the -5p form and the less abundant -3p form which are then processed in the cytoplasm to mature miRs (Duchaine and Fabian, 2019). The chicken genome is predicted to encode ∼900 miRs (Burt, 2005; Wallis et al., 2004) however only a few miRs have been studied in detail regarding their role in regulating viral replication. Previous studies from our laboratory also demonstrated that chicken miR-26a regulated APMV-1 replication by two independent mechanisms. MiR-26a was demonstrated to directly target APMV-1 L gene; miR-26a was also demonstrated to regulate Interferon induced helicase 1 (IFIH1), also known as Melanoma differentiation antigen 5 (MDA5), a key sensor of dsRNA replicative intermediates formed during APMV-1 replication (A and M, 2026). We previously demonstrated that chicken miR-27b-5p can inhibit the levels of APMV-1 P RNA (Mears and Bakre, 2024). Perfect sequence complementarity between the miR-27b-5p seed site and a highly conserved binding site in the APMV-1 P transcript of the 2018 vNDV strain from California (CA18) caused degradation of the APMV-1 P RNA upon transient overexpression of miR-27b-5p (Mears and Bakre, 2024).

The central hypothesis of this manuscript was to test if encoding gga-miR-27b within the LaSota vaccine strain impacted viral replication and expression of both viral and host genes. The entire gga-miR-27b construct was introduced into the LaSota backbone to ensure that miR processing and expression closely follow the canonical miR biogenesis pathway and produce both gga-miR-27b-5p and gga-miR-27b-3p miRs. The data demonstrate that gga-miR-27b expression from within the LaSota backbone can modulate viral replication via viral targeting without resulting in mortality of embryonating chicken eggs (ECE), which confirms that the recombinant virus remained non-virulent. These data demonstrate that encoding miR cassettes in vaccine platforms can be used to regulate vaccine virus replication. Secondly, in addition to inducing humoral and cellular immunity, these miR encoding vaccine platforms can provide an additional mechanism of controlling viral replication.

## Materials and methods

### Cells and viruses

Chicken fibroblast cells (DF-1) (CRL-3586) were obtained from the American Type Culture Collection (ATCC, VA, USA) and grown in DMEM containing high glucose, 1X antibiotic-antimycotic mix, 1 mM Sodium pyruvate, 1X Glutamax and 10 % heat inactivated fetal bovine serum (FBS)(Biowest, USA) (referred to as complete DMEM medium hereafter) at 39°C with 5 % CO_2_ as recommended by ATCC. The cells were confirmed to be mycoplasma free using mycoplasma specific primers.

The LaSota strain of APMV-1 (USNPRC repository) was grown and harvested from ECEs as described previously (Msoffe et al., 2019). Viral titers were determined as 50 % egg infectious doses (EID50) and hemagglutination (HA) assays were performed according to the WOAH manual (OIE, 2021).

### Design and rescue of rLS-miR-27b and rLS-Cel67 viruses

The 97 bp stem-loop sequence for *gga*-miR-27b (miR-27b) was designed with primer binding sites and 15-bp extensions for cloning, and was synthesized as a gBlock (Integrated DNA Technologies, USA). A gBlock was also synthesized using the *C. elegans* miR Cel-67 (miR-Cel67) sequence with the same flanking primer and extension sequences as a non-specific control. The Cel-miR-67 miR has no known targets in the chicken genome, has the length equivalent to the miR-27b; no sequence complementarity and thus functions as an appropriate control. The gBlocks were cloned into a linearized pLS-GFP vector backbone using the In-Fusion cloning master mix as previously described (Takara Biotech, USA) (Supplementary material 1) (He et al., 2020). Stbl3 cells (Invitrogen, USA) were immediately transformed as per manufacturer’s protocol and plated on LB-Carbenicillin agar plates (100µg/ml) then incubated overnight at 37°C. Transformants were screened by colony PCR using oligonucleotides Uni-miR-F (5’- AGTTTAGTTGATAGTTGTAGC-3’) and Uni-miR-R (5’-GCTTAGTACACGGTAGTTA-3’) with the following PCR conditions: Initial denaturation at 95°C for 2 min, 25 cycles of 95°C for 30 seconds, 56°C for 30 seconds, and 72°C for 1 min followed by final elongation at 72°C for 5 min before storage at 4°C. Amplicons were electrophoresed on a 3 % agarose gel in 5 mM Lithium acetate buffer at 2.5V/cm for 1 hour. Correct sized amplicons were excised from the gel, purified, and then sequenced using Sanger sequencing to validate gBlock insertion into the vector. Sequence validated constructs and helper plasmids encoding APMV-1 N, P, and L genes were grown and plasmids were isolated using NucleoBond Xtra-Midi protocol (Machery-Nagel, USA) to obtain endotoxin-free transfection grade plasmid preparations. Plasmids were quantified using Nanodrop 2000 (Thermofisher USA).

### Recombinant APMV-1 rescue

Human epithelial cells (Hep-2) grown to 95-100 % confluency in a 6 well plate in DMEM with 10 % FBS were washed with serum-free DMEM and then infected with a replication deficient vaccinia virus expressing T7 RNA polymerase (MVA-T7) (multiplicity of infection = 3) for 1 hour. Although, not expected, because miR expression in the APMV-1 backbone could potentially alter replication and viral fitness, transfections and virus rescues were carried out under BSL-3 enhanced conditions. Plasmids constructed above for miR-27b, miR-Cel67, and the GFP control (pLS-miR-27b, pLS-miR-Cel67, and pLS-GFP respectively) and helper plasmids encoding N, P, and L were co-transfected into the MVA-T7 infected Hep2 cells using Lipofectamine 3000 as per manufacturer’s protocol. Infected and transfected cells were subjected to three freeze-thaw cycles 48 hours post transfection and used to inoculate ECEs. Five ECEs (9-10 days old) were inoculated with each lysate in the allantoic cavity to amplify the rescued virus. ECEs were monitored for viability for 5 days and allantoic fluids (AFs) were harvested from each ECE and tested for viral protein presence using HA assays. AFs with similar HA titers were pooled, clarified by centrifugation at 1200 rpm for 10 min at 4°C, then frozen in single-use aliquots. For subsequent passages, AFs were diluted 1:1000, treated with 1X antibiotic-antimycotic mix for 30 min before inoculation into the allantoic cavity of 9-10 day old ECEs. Virus stocks from egg passage 3 were used for all *in vitro* experiments. Viral titers were determined by EID_50_ as per standard procedure.

### Recombinant virus replication kinetics

DF-1 cells in 6 well plates were incubated overnight in complete DMEM medium as described. Cells were washed once with serum-free DMEM and then infected in biological triplicates with rLS-27b, rLS-Cel67, or rLS-GFP using a multiplicity of infection (MOI) of 1 for 1 hour before the inoculum was removed and replaced with complete DMEM. Plates were subjected to three freeze-thaw cycles at 0, 12, 24, 48, 72, 96, and 120 hours post infection (hpi). Cell lysates were used to determine HA titers or to isolate total RNA for qRT-PCR for APMV-1 M, L and other host genes.

### Total RNA isolation and qRT-PCR

Total RNA from cell lysates or viral stocks was isolated using Trizol LS reagent as per manufacturer’s protocol . Equal amounts of quantified RNA were reverse transcribed using Maxima H minus reverse transcriptase (ThermoFisher Scientific, USA). Real time PCR reactions for viral genes (APMV-1 M and L) or host genes contained 1 µl of 1:10 diluted cDNA, 1X Luna Universal SYBR Green master mix (New England Biolabs, USA), gene specific forward and reverse oligos (250 nM final concentration) and were amplified using the following program: initial denaturation at 95°C for 1 min, 40 cycles of 95°C for 15 seconds, 56°C for 30 seconds, 60°C for 30 seconds followed by melt curve analysis. Chicken 18S rRNA was used as a reference gene. Fold changes were calculated using the ΔΔCt method (Bustin et al., 2025; Bustin et al., 2009). For miR expression analysis, reactions were performed as above with miR specific forward oligo, a universal reverse oligo and annealing temperature of 43°C instead followed by melt curve analysis and standard curves. All miR qRT-PCRs yielded single peaks in melt curves with melt temperatures above 70°C indicating amplification of a single species.

### APMV-1 P editing analysis

A 436 bp amplicon flanking the P editing site was amplified from first strand cDNAs of rLS-GFP, rLS-Cel67, and rLS-27b viruses across 10 viral passages (from passage 3 through 12) with primers LAF (5’-GCAGCATGGGAGAA-3’) and LAR1 (5’-CTGATAGTTGTGACTCCTC-3’) using Q5 2X master mix (NEB, USA) under the following conditions: initial denaturation at 98°C for 1 min, 25 cycles of 95°C for 10 seconds, annealing at 70°C for 30 seconds and extension at 72°C for 30 seconds as per manufacturer recommendations. PCR products were resolved on a 1.5 % gel in 1X TAE buffer, amplicons of the correct size were excised from the gel, purified, quantified, and then sequenced using the Illumina amplicon sequencing kit.

### Whole genome sequencing of rLS-27b, rLS-Cel67 and rLS-GFP viruses

Total RNA from stocks of individual passages of rLS-GFP, rLS-Cel67, and rLS-miR-27b was isolated using the Trizol LS as per manufacturer’s protocol. Quantified RNA was depleted of chicken rRNA using a previously developed host depletion workflow (Bakre et al., 2023; Parris et al., 2022), reverse transcribed and subjected to sequence independent single primer amplification (SISPA) (Parris et al., 2022). Using the Illumina DNA Prep kit, amplified libraries underwent tagmentation and barcoding with Illumina DNA/RNA UD Indexes. NGS libraries were quantified and quality checked using Qubit (ThermoFisher) and TapeStation (Agilent) respectively, and pooled at 1 nM final concentration. The pooled library was denatured and loaded at 100 pM alongside a 2 % spike (by volume) of a phiX control (60 pM) and finally sequenced on an Illumina MiSeq i100 with a 25 M Reagent 600 cycle kit. BCL Convert from DRAGEN (v4.3.13, Illumina) was used to demultiplex the reads, remove barcode adapters, initially filter out low quality reads, and fastq generation.

### Statistical analysis

Error bars represent ± standard deviation of three or more biological replicates. Statistical comparisons were performed using one way or two-way ANOVA as appropriate with a repeated measures / mixed effects model with Tukey’s / Dunnett’s post hoc analysis in GraphPad Prism version 11.0.0 (84). Statistical significance is indicated as *=*p*<0.05, **=*p*<0.01, ***=*p*<0.005 and ****=*p*<0.0001. Details on individual methods are provided in figure legends.

## Results

### Generation of recombinant LaSota viruses encoding miRs

The well-characterized full-length cDNA clone pLS-GFP containing the complete antisense genome of the APMV-1 LaSota vaccine strain with green fluorescent protein (GFP) between the P and M genes served as the vector for generation of new full-length cDNA clones (Yu et al., 2023; Zhao et al., 2013; Zhao et al., 2015). GFP was replaced with an independent transcription unit encompassing the complete pre-miR sequence from either chicken miR-27b or *C. elegans* miR-67, noncoding regions for efficient transcription, and extra nucleotides to abide by the paramyxovirus rule of six, between APMV-1 gene start and gene end sequences (Fig. 1). The central hypothesis was that the gga-miR-27b construct should generate both miR-27b-5p and miR-27b-3p while the Cel-67 construct is expected to produce cel-miR-67-5p and cel-miR-67-3p miRs. The full pre-miR sequence cassette was also included to ensure that miR expression and processing closely followed the canonical miR biogenesis pathway. Sanger sequencing verified constructs and helper plasmids were used to rescue recombinant viruses through an infection/co-transfection method, followed by amplification in ECEs. Because the impact of miR expression on LaSota replication and virulence was unknown, all virus (rLS-GFP, rLS-miR-Cel67 and rLS-miR-27b) rescue was performed in BSL-3 as mandated by institutional biosafety. All recombinant viruses were passaged a total of 10 times in ECEs to evaluate insert stability, with passage 3 serving as the primary stock used for experiments throughout this manuscript.

**Fig. 1:**
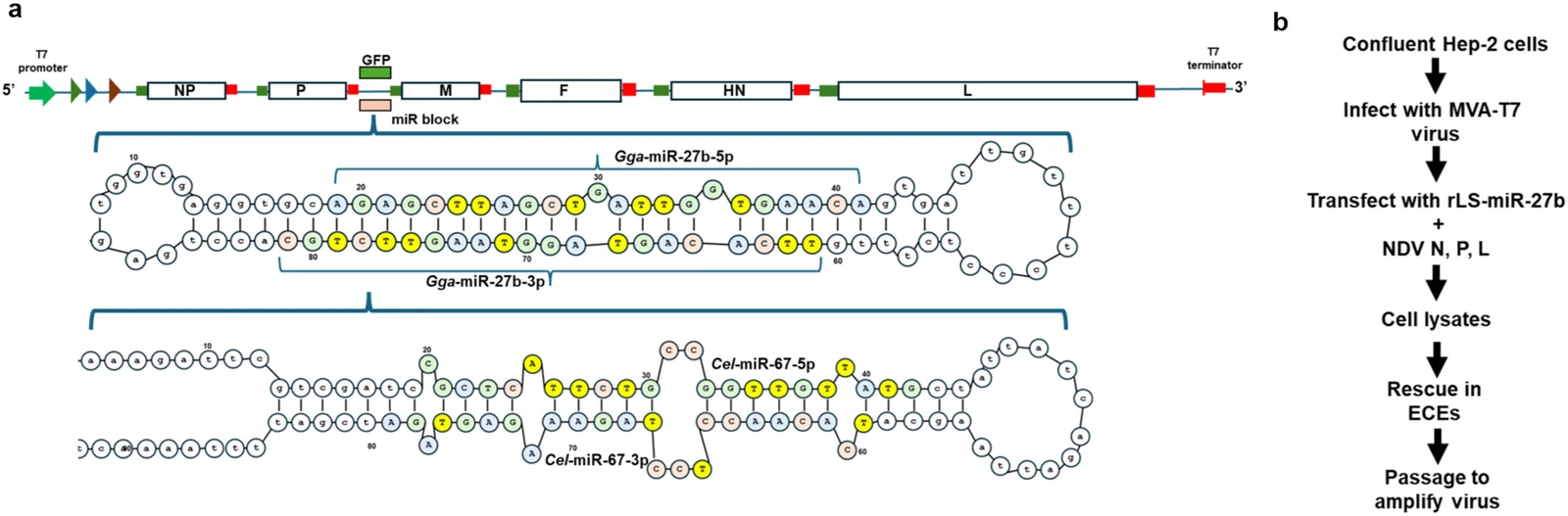
Design of rLS-miR-27b vaccine candidate. (a) Strategy to clone and express chicken miR -27b or Cel-miR-67 in the LaSota backbone is shown. Colored bases indicate the -5p and -3p mature miRs encoded by gga-miR-27b or Cel-miR-67. (b) Workflow to rescue recombinant LaSota viruses expressing chicken gga-miR-27b or Cel-miR-67 is shown.

### rLS-miR-27b exhibits no egg toxicity

A mean death time (MDT) assay was used to determine if endogenous expression of *gga*-miR-27b altered the *in ovo* virulence of the APMV-1 LaSota strain. The MDT of the recombinant LaSota viruses rLS-GFP, rLS-Cel67, and rLS-miR-27b were all >168 hpi, with no ECE death recorded. These data demonstrate that endogenous expression of *gga*-miR-27b did not increase virulence of the LaSota strain *in ovo*.

### miR-27b reduces viral transcripts without reducing overall viral titer

APMV-1 P is critical to inhibition of the cellular interferon response via production of V and W proteins, and is part of the viral polymerase complex (Huang et al., 2003; Park et al., 2003). Since we previously demonstrated that chicken miR-27b-5p could bind and reduce APMV-1 P transcript levels, it was expected that constitutive expression of miR-27b-5p from the miR-27b cassette in rLS-miR-27b might also impair viral replication. To determine if insertion of the miR-27b cassette into the LaSota genome affected viral replication in ECE, virus titers were compared from the passage 3 stocks. There was no statistical difference in the peak titer between rLS-miR-27b compared to the rLS-GFP and rLS-miR-Cel67 control viruses (Fig. 2a).

**Fig. 2:**
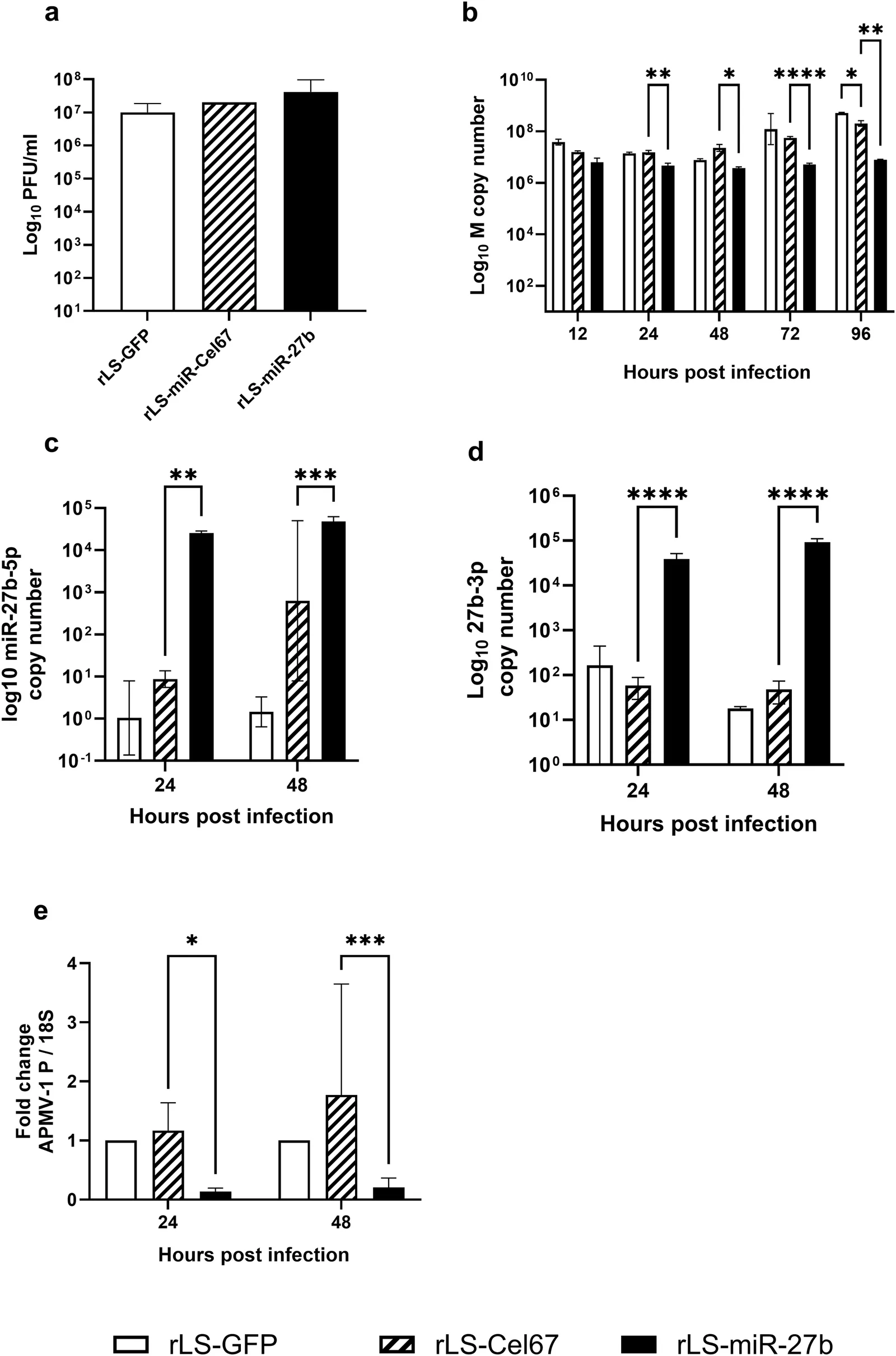
Kinetics of rLS-miR-27b growth (a) Log10 virus titers (PFU/mL) of rLS-GFP (clear bar), rLS-miR-Cel67 (hatched bar) and rLS-miR-27b (filled bar) are graphed. Error bars indicate ±SEM from three replicates. All data obtained from egg passage 3 virus stocks (b) Log10 matrix (M) gene copies from DF-1 cells infected with rLS-GFP (clear bar), rLS-miR-Cel67 (hatched bar) and rLS-miR-27b (filled bar) are plotted. Error bars indicate ±SD from three biological replicates. Groups were compared by repeated measures 2-way ANOVA with Geisser-Greenhouse correction and post-hoc Tukey’s multiple comparisons test, confidence level set to 0.05. *=p<0.05, **=p<0.005,****=p<0.0001.(c) Log10 copy numbers for mature miR-27b-5p and miR-27b-3p (d) from rLS-miR-Cel67 (hatched bar) and rLS-miR-27b (filled bar) are graphed at 24 and 48 hpi. Error bars indicate ±SD from three biological replicates. Groups were compared by repeated measures 2-way ANOVA with no correction and post-hoc Tukey’s multiple comparisons test with a single pooled variance, confidence level set to 0.05. **=p<0.005, ***=p<0.0005, ***=p<0.0001. (e) Fold change in APMV-1 P levels following infection with rLS-GFP (open bar), rLS-miR-Cel-67 (hatched bar) or rLS-miR-27b (closed bar) are graphed. Groups were compared by mixed effects model with the Geisser-Greenhouse correction model and post-hoc Tukey’s multiple comparison test with single pooled variance and confidence interval set to 0.05. *=p<0.05.

To further evaluate differences in the viral replication kinetics, DF-1 cells were infected with rLS-miR-27b, rLS-miR-Cel67, and rLS-GFP viruses at an MOI=1, then total RNA was harvested at different timepoints post-infection and evaluated for viral gene transcripts. While there was an increase in M gene transcripts over time from the rLS-GFP and rLS-miR-Cel67 viruses, the amount of M gene transcripts from rLS-miR-27b infection remained unchanged over time (Fig. 2b). To demonstrate that the miR-27b cassette in rLS-27b was transcribed, copy numbers of mature miR-27b-5p and miR-27b-3p were determined from rLS-Cel67, rLS-GFP or rLS-27b infected cells (at both 24 and 48 h.p.i) by absolute qRT-PCR using 10-fold dilutions of the miR-27b construct to generate standard curves as per MIQE guidelines. The data clearly demonstrated 4-4.5 log increase in miR-27b-5p expression relative to rLS-Cel-67 (Fig. 2c) and 2-2.5 log increase for gga-miR-27b-3p (Fig. 2d). These data confirm that both miR-27b-5p and miR-27b-3p are expressed from the rLS-miR-27b virus. Additionally, there was a significant reduction in P gene transcripts from rLS-miR-27b infected cells compared to the rLS-GFP and rLS-miR-Cel67 controls at 24 and 48 hpi (Fig. 2e). Together, these data suggest that expression of *gga*-miR-27b from within the LaSota genome does not impair overall viral titer *in vitro* and shows an inverse correlation with increased expression of miR-27b-5p as originally hypothesized. Because gga-miR-27b and Cel-miR-67 lack any sequence similarity, these differences between rLS-miR-Cel67 and rLS-miR-27b phenotypes are likely driven by construct expression.

### rLS-miR-27b modulates interferon expression

As shown in Fig. 2e, expression of *gga*-miR-27b from within the LaSota genome reduced APMV-1 P gene transcripts. Since APMV-1 P and its products V and W are involved in inhibition of interferon signaling (Huang et al., 2003; Qiu et al., 2016; Sun et al., 2019; Nan et al., 2021), we hypothesized that the reduction of P transcripts within rLS-miR-27b infected cells would result in increased host interferon responses. Because, all viral rescues and infections were performed in BSL-3 and our current inactivation protocols prevent transferring lysate or supernatants for protein level analysis to BSL-2, impact of miR-27b-5p expression on the interferon expression was investigated at the transcript level. Total RNA harvested from DF-1 cells infected with rLS-miR-27b, rLS-miR-Cel67, and rLS-GFP viruses (MOI=1) at 24 and 48 hpi was evaluated by qRT-PCR for expression of type I and III interferons (*IFN-α*, IFN-β and *IFN-λ* respectively) and interferon stimulated genes (ISGs) MX1 (MX dynamin GTPAse 1). There was a significant increase in IFN-α transcripts at 24 hpi that returned to near baseline levels by 48 hpi (Fig. 3a). Expression of IFN-β did not change at 24 hpi but showed a slight increase at 48 hpi; this increase was not statistically significant (Fig. 3b). Expression of IFN-λ transcripts were not significantly different between viruses at 24 hpi, but increased significantly at 48 hpi (Fig. 3c) in rLS-27b infected cells. Expression of MX1 was elevated slightly at 24 hpi but returned to baseline by 48 hpi (Fig. 3d). This data, combined with the demonstrated reduction of P gene transcripts by *gga*-miR-27b suggests that the targeted reduction of P, V, and W genes potentially blocks their inhibition of host interferon responses, therefore allowing infected cells to mount a stronger innate response to rLS-miR-27b compared to the rLS-miR-Cel67 and rLS-GFP control viruses.

**Fig. 3:**
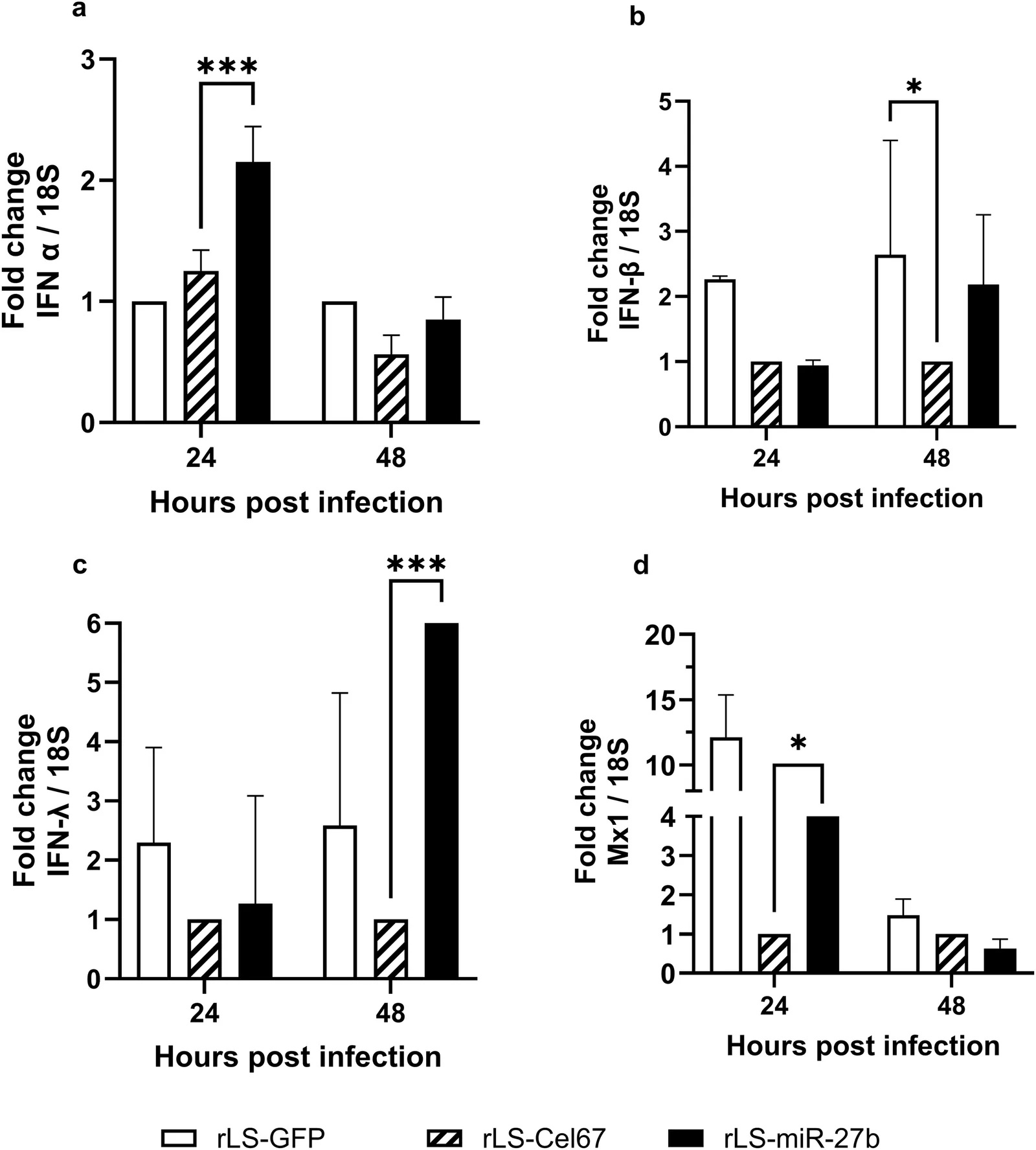
rLS-miR-27b virus regulates host IFN response via APMV-1 P suppression. (a) Fold change in IFN-α expression following infection with rLS-GFP (open bar), rLS-miR-Cel-67 (hatched bar) or rLS-miR-27b (closed bar) are graphed. (b) Fold change in IFN-β expression following infection with rLS-GFP (open bar), rLS-miR-Cel-67 (hatched bar) or rLS-miR-27b (closed bar) are graphed. (c) Fold change in IFN-λ expression following infection with rLS-GFP (open bar), rLS-miR-Cel-67 (hatched bar) or rLS-miR-27b (closed bar) are graphed. Error bars indicate ±SD of eight independent replicates for each time point. Groups were compared by repeated measures 2-way ANOVA or mixed effects model with Geisser-Greenhouse correction and post-hoc Tukey’s multiple comparisons test, confidence level set to 0.05. *=*p*<0.05, **=*p*<0.005, ****=*p*<0.0001.(d) Fold change in Mx1 expression following infection with rLS-GFP (open bar), rLS-miR-Cel-67 (hatched bar) or rLS-miR-27b (closed bar) are graphed. Error bars indicate ±SD of three independent replicates for each time point. Groups were compared by repeated measures 2-way ANOVA or mixed effects model with Geisser-Greenhouse correction and post-hoc Dunnet’s multiple comparisons test with a single pooled variance and confidence level set to 0.05. *=*p*<0.05, **=*p*<0.005, ****=*p*<0.0001.

### miR-27b expression from rLS-miR-27b does not change P/V transcript editing frequency

Insertion of nucleotides by the viral RNA polymerase within the P gene produces the transcripts for P, V, and W, with identical N-termini, but different C-termini. Because these 3 transcripts are produced by the same viral gene, we hypothesized that miR-27b-mediated reduction of P may also reduce V transcripts, contributing to the reduced interferon inhibition slowing viral replication. To evaluate this, a 436 bp region (2031-2466) flanking the P gene RNA editing site (nucleotides 2280-2285) in APMV-1 strain LaSota was amplified using a proof-reading enzyme from rLS-GFP, rLS-miR-Cel67, and rLS-miR-27b RNA across 10 viral passages (P3-P12), then sequenced. As expected, most reads mapped to nucleotides 2031-2466 of the P gene. These were extracted, aligned to the APMV-1 LaSota genome and analyzed for the abundance of the P motif (AAAAAG (A_5_G)) and V motif (AAAAAGG (A_5_GG)) across samples and passages. At passage 3, rLS-Cel67 produced only 5882 P-specific reads, limiting meaningful comparison with rLS-GFP or rLS-miR-27b. Similarly, at passage 12, rLS-Cel67 yielded only ∼11000 reads, none of which mapped to the P gene, again preventing comparisons with the other two viruses. Low read counts were also observed for rLS-GFP at passage five, seven and nine. Considering that variations in read counts are well documented due to differential PCR amplification, quantification and index bias, these deviances likely reflect experimental error rather than construct instability. This is further supported by comparable high read counts for all three viruses across other passages. (Table 1). For all three viruses, across passage 4 through 11, the percentage of reads covering the P and V motif regions were generally comparable with only minor variations. As expected, data showed that the A_5_G P motif was more abundant than the A_5_GG V motif in all three viruses across passages as indicated by the P/V ratio. The consistent P/V motif ratio between rLS-miR-Cel67 and rLS-miR-27b across passages suggests that while gga-miR-27b-5p reduces P transcripts, it does not impact the amount of viral transcript editing in recombinant virus stocks.

**Table 1:** Relative abundance of P and V transcript motifs in different passages of rLS-GFP, rLS-Cel67 and rLS-27b viruses is tabulated. Data for rLS-Cel67 and rLS-27b viruses are indicated in bold and italics respectively. Percentages of total reads are indicated in parentheses. P = passage number. NA = Not available.

Passage	rLS Virus	P motifs	V motifs	Ratio (P/V)	Total Reads
3	GFP	1112 (0.12 %)	477 (0.05 %)	2.3	940099
	miR-Cel67	68 (1.16 %)	12 (0.20 %)	5.6	5882
	miR-27b	798 (0.07 %)	402 (0.04 %)	1.98	1076815

4	GFP	973 (0.08 %)	523 (0.04 %)	1.86	1245356
	miR-Cel67	1428 (0.09 %)	774 (0.05 %)	1.84	1636584
	miR-27b	784 (0.09 %)	433 (0.05 %)	1.81	910566

5	GFP	201 (1.73 %)	38 (0.33 %)	5.28	11631
	miR-Cel67	480 (0.25 %)	177 (0.09 %)	2.7	191332
	miR-27b	991 (0.08 %)	531 (0.04 %)	1.86	1255026

6	GFP	1316 (0.08 %)	717 (0.05 %)	1.83	1580825
	miR-Cel67	1003 (0.08 %)	529 (0.04 %)	1.89	1240120
	miR-27b	722 (0.10 %)	363 (0.05 %)	1.98	733124

7	GFP	1396 (4.00 %)	341 (0.98 %)	4.09	34889
	miR-Cel67	1346 (0.10 %)	703 (0.05 %	1.91	1414671
	miR-27b	778 (0.09 %)	413 (0.05 %)	1.88	860716

8	GFP	1479 (0.09 %)	717 (0.04 %)	2.06	1723743
	miR-Cel67	1646 (0.10 %)	974 (0.06 %)	1.68	1700293
	miR-27b	879 (0.09 %)	500 (0.05 %)	1.75	1025173

9	GFP	1161 (1.94 %)	132 (0.22 %)	8.7	59931
	miR-Cel67	2232 (0.09 %)	1138 (0.05 %)	1.96	2368874
	miR-27b	806 (0.08 %)	419 (0.04 %)	1.92	981237

10	GFP	1657 (0.08 %)	834 (0.04 %)	1.98	2105067
	miR-Cel67	1371 (0.09 %)	633 (0.04 %)	2.16	1580938
	miR-27b	751 (0.07 %)	366 (0.04 %)	2.05	1020572

11	GFP	1657 (0.07 %)	834 (0.04 %)	5.28	2239035
	miR-Cel67	1371 (0.11 %)	633 (0.05 %)	2.34	1283752
	miR-27b	751 (0.09 %)	366 (0.05 %)	2.04	791730

12	GFP	1242 (0.09 %)	721 (0.05 %)	1.72	1454524
	miR-Cel67	NA	NA	NA	11628
	miR-27b	138 (0.06 %)	43 (0.02 %)	3.21	226384

## Discussion

MiRs perform important roles in regulating cellular pathways, and multiple studies demonstrate a clear role for miRs both in viral replication and regulation of the immune response to viral infection (Chen et al., 2020; Chowdhury et al., 2024; Teng et al., 2023; Yao and Nair, 2014; Mears and Bakre, 2024; Li and Zheng, 2020; Buck et al., 2010). MiRs of the miR-27b family have been shown to have a role regulating viruses in both chickens and other hosts. In chickens, miR-27b-3p has been shown to be induced in the trachea during infection with a highly pathogenic avian influenza H5N1 strain (Kang et al., 2023), and it has been shown to have an important role in regulating the replication of infectious bursal disease virus (IBDV) (Duan et al., 2020). Beyond poultry, miR-27b exhibits antiviral functions by inhibiting replication of mouse cytomegalovirus (MCMV) (Buck et al., 2010), transmissible gastroenteritis virus (TGEV) (Zhao et al., 2016), and hepatitis C virus (HCV) (Sakurai et al., 2019).

Host microRNAs have been demonstrated to interact with RNA viral genomes and inhibit viral gene translation (Trobaugh et al., 2014) or enhance viral replication (Jopling et al., 2005; Shimakami et al., 2012). In case of human hepatitis C virus (HCV), the interaction with miR-122 is critical for viral replication. Inhibition of miR-122 through antagonists dramatically reduced HCV titers in chimpanzees and humans (Jopling et al., 2005; Thibault and Wilson, 2013) and led to the development of a first anti-miR targeted treatment for HCV and development of a next generation antagomir RG-101 which is currently under phase II clinical trials (Bonneau et al., 2019).

Previously, transient upregulation of *gga*-miR-27b-5p was demonstrated to reduce APMV-1 P gene expression via direct sequence complementarity (Mears and Bakre, 2024). The impact of constitutive miR-27b expression on APMV-1 replication was explored in this manuscript by expressing the entire *gga*-miR-27b from the APMV-1 LaSota backbone in the well characterized intergenic region between the P and M genes. This pre-miR construct was expected to express both gga-miR-27b-5p (the major miR) and gga-miR-27b-3p (the minor variant) and allows processing and expression in the native miR biogenesis pathway. The recombinant virus, rLS-miR-27b, expressing the *gga*-miR-27b cassette, in addition to control viruses (Fig. 1), was rescued by standard APMV-1 reverse genetic methods (Yu et al., 2023) in BSL-3 containment to address concerns about potential gain of function. Because the parental rLS-GFP virus carries a much larger insert (∼720 bp) compared to the rLS-miR-27b virus (97 bp), a control virus rLS-Cel67 that expresses a comparably sized miR from *C. elegans* (*cel*-miR-67 (∼77 bp)) was also rescued and used as a control in all experiments. The Cel-miR-67 sequence bears no sequence complementarity with AMPV-1 P gene and thus functions as a non-targeting control. Previous data demonstrate that differences in replication kinetics of rLS-GFP and wild type LaSota were small and insignificant (Hu et al., 2018). Replication of the rLS-miR-27b virus in ECEs was comparable to the controls (Fig. 2a); however, replication in DF-1 cells was altered over time (Fig. 2b). Because repression of the APMV-1 P gene by miR-27b-5p was previously demonstrated, we hypothesized that slower replication of rLS-miR-27b was potentially due to suppression of P gene expression, as P is a critical cofactor in the viral polymerase complex. The impact of miR-27b on P gene expression was demonstrated by a significant reduction of P transcripts from rLS-miR-27b infected cells compared to controls (Fig. 2c). This suggests that over expression of gga-miR-27b inversely correlated with reduction in viral P transcript and possibly led to significant reduction in the replication kinetics of the recombinant virus. These differences are likely because ECEs provide multiple cell types for infection, activated amino acid biosynthesis pathways necessary for viral assembly and multiple proteases that assist both viral infection and egress (Yang et al., 2024). The replication in DF-1 cells is likely reduced because DF-1 cells have increased expression of interferon inducible proteins with transmembrane domains (IFITM), a key anti-viral molecule. Knockout of IFITM has been validated to lead to substantial increase in titers for both low pathogenicity influenza A virus (LAIV) and APMV-1 (Samy et al., 2025). Further, DF-1 cells do not express the variety of proteases typically associated with the allantoic fluid in ECEs and this limits viral infectivity and replication and expression of exogenous proteases increased viral replication (Chungu et al., 2021). This likely explains the reduced replication of rLS-miR-27b virus in DF-1 cells and higher titers in ECEs.

APMV-1 P gene expression is known to antagonize host IFN response by a variety of mechanisms, including by the production of the V and W transcripts (Park et al., 2003; Alamares et al., 2010; Wang et al., 2019). Here, data demonstrate that *gga*-miR-27b-5p overexpression correlated with reduction of the APMV-1 P and also with increased expression of *IFN-α, IFN−β* and *IFN-λ* transcripts (Fig. 3a and 3b). Changes in IFN-α or IFN−β protein level could not be investigated because all viral rescues were performed in BSL-3 containment and our current inactivation protocols prohibit removal of cell lysate or supernatants for protein analysis. These data thus reflect preliminary evidence at the transcriptional level. However, while *gga*-miR-27b-5p reduces P transcripts overall, P/V ratios were unchanged (Table 1), suggesting that as shown previously, the upregulation of *IFN-α, IFN-β* and *IFN-λ* transcripts is due to the impact of miR-27b on AMPV-P and not V.

It is important to note that since each miR can potentially regulate hundreds of genes or pathways, the above observations are likely one of many other pathways that regulate APMV-1 replication and life cycle. In addition to direct antiviral impacts, miRs also play a role in regulating cellular pathways and immune responses. Among host genes, miR-27b has been demonstrated to regulate multiple cellular genes, such as carbonic anhydrase X (*CA10*) (Chung et al., 2022), and transcription factors such as c-Jun, peroxisome proliferator-activated receptor alpha (*PPARα*), and hepatocyte nuclear factor 4 alpha (*HNF4α*) (Desrochers et al., 2022). Further comparison of predicted miR-27b-5p or -3p targets with genes deregulated during APMV-1 infection *in vivo* from other studies demonstrated that several key genes induced or repressed during infection are conserved miR-27b targets (Supplementary Table 1). Previously, the upregulation of *gga*-miR-27b-3p, the other miR from the miR-27b construct was demonstrated to induce expression of *NF-κβ, IRF3* and *IFN-b* by targeting the suppressors of cytokine signaling 3 and 6 (*SOCS3* and *SOCS6*) molecules. Consequently, it was shown that inhibition of gga-miR-27b-3p, facilitated IBDV replication by suppressing NF-kβ, *IRF3* and *IFN-β* and a concomitant increase in *SOCS3* and *SOCS6* (Duan et al., 2020). Together, these data highlight the important balance of miRs and host innate immune responses that can be exploited for poultry vaccine improvement.

The significant genetic diversity and number of genotypes of AMPV-1 causes significant issues for vaccine development to produce widely cross-protective ND vaccines. Genotype mismatch between current ND vaccines and outbreak strains can often lead to poor protection. By expressing a highly conserved host miR that targets a conserved viral motif independent of genotype in live vaccines, it is possible to reduce viral gene expression and downstream poultry morbidity and mortality, and economic damage to the poultry producer due to outbreak strains. Previous work utilizing miR recognition elements (MREs) or expression of some host miRs with influenza viruses has demonstrated potential for reducing viral burden and subsequent mortality in animal models (Perez et al., 2009; Langlois et al., 2013; Li et al., 2015; Waring et al., 2018). Because viruses often share conserved host pathways for their replication and egress from host cells during infection, using miRs that target these conserved genes and pathways may be useful to develop vaccine candidates that have a broader spectrum of activity against multiple avian pathogens. The stability of miRs in biofluids is well documented (Mall et al., 2013) thus it is expected that miRs produced from rLS-miR-27b and similar constructs would be present in the cellular milieu for a duration following vaccination. Since the LaSota vaccine is often applied as a coarse spray at hatch and boosted 21 days later, administering vaccines including stable host miRs might bolster protection after waning of maternal derived antibody. Secondly, miRs are also exported out of cells via exosomes (Neerukonda et al., 2019; Nath Neerukonda et al., 2019; Hong et al., 2021; Kim et al., 2025). Exosomes from rLS-miR-27b infected cells have the potential to deliver the miR-27b-5p and miR-27b-3p cargo to neighboring recipient cells and induce an anti-viral state. This has been shown for other miRs and other pathogens (Mohammadinasr et al., 2024; Yin et al., 2023; Truong et al., 2023; Zhou et al., 2018).

To summarize, this is the first report of a miR expressed from the LaSota vaccine backbone of APMV-1 that demonstrates that host miRs can be utilized to regulate viral gene expression in vaccine platforms. These preliminary data in this manuscript thus suggest a novel approach to improve APMV-1 vaccines. Further safety and immune efficacy testing *in vivo* are warranted to evaluate this novel platform.

## Declarations

The authors have no commercial conflicts to declare.

## Ethics approval and consent to participate

Not applicable.

## Consent for publication

All authors agree to the content of the manuscript and consent for publication if accepted.

## Funding

This work was supported by the U.S. Department of Agriculture-Agricultural Research Service under project number 6040-32000-082-00D.

## Author contributions

AB conceived and designed the study, administered the project, performed experiments and data analysis, wrote the initial draft and revisions and approved final version of the manuscript. TS designed sequencing strategy project, performed experiments and sequenced samples and assisted with analysis, revised the draft and final version of the revised manuscript for scientific authenticity MCM designed viral constructs, rescued recombinant rescue viruses, wrote the initial draft and revisions and approved final version of the manuscript.

## Disclosures

The authors declare no conflict of interest. The funders had no role in study design; collection, analyses, or interpretation of data; writing of the manuscript; or decision to publish the results.

## Data Availability

The data that support the findings will be made available in AgDataCommons before publication or from the corresponding author upon request. Supplemental data is provided.

## Footnotes

Supplementary material associated with this article can be found, in the online version, at doi:10.1016/j.psj.2026.107893.

## References

[bib0002] Alamares J.G. (2010). The interferon antagonistic activities of the V proteins from two strains of Newcastle disease virus correlate with their known virulence properties. Virus Res..

[bib0003] Bakre A., Kariithi H.M., Suarez D.L. (2023). Alternative probe hybridization buffers for target RNA depletion and viral sequence recovery in NGS for poultry samples. J. Virol Methods.

[bib0001] Bakre A., Mears M.C. (2026). Chicken microRNA 26a-5p regulates replication of Newcastle disease virus by direct targeting of the viral polymerase. Virol J..

[bib0004] Bello M.B. (2018). Diagnostic and vaccination approaches for Newcastle disease virus in poultry: the current and emerging perspectives. Biomed. Res. Int..

[bib0005] Bonneau E. (2019). How close are miRNAs from clinical practice? A perspective on the diagnostic and therapeutic market. EJIFCC.

[bib0006] Buck A.H. (2010). Post-transcriptional regulation of miR-27 in murine cytomegalovirus infection. RNA.

[bib0007] Burt D.W. (2005). Chicken genome: current status and future opportunities. Genome Res..

[bib0008] Bustin S.A. (2009). The MIQE guidelines: minimum information for publication of quantitative real-time PCR experiments. Clin. Chem..

[bib0009] Bustin S.A. (2025). MIQE 2.0: revision of the minimum information for publication of quantitative real-time PCR experiments guidelines. Clin. Chem..

[bib0010] Chen X. (2020). Epigenetic regulation by non-coding RNAs in the Avian Immune system. Life (Basel).

[bib0011] Chowdhury D. (2024). Role of miRNA in highly pathogenic H5 Avian Influenza Virus infection: an emphasis on cellular and chicken models. Viruses.

[bib0012] Chung K.M. (2022). CA10 is associated with HBV-related hepatocarcinogenesis. Biochem. Biophys. Rep..

[bib0013] Chungu K. (2021). Establishment of a genetically engineered chicken DF-1 cell line for efficient amplification of influenza viruses in the absence of trypsin. BMC Biotechnol..

[bib0014] Desrochers G.F. (2022). microRNA-27b regulates hepatic lipase enzyme LIPC and reduces triglyceride degradation during hepatitis C virus infection. J. Biol. Chem..

[bib0015] Dimitrov K.M. (2017). Newcastle disease vaccines-A solved problem or a continuous challenge?. Vet. Microbiol..

[bib0016] Dimitrov K.M. (2019). Updated unified phylogenetic classification system and revised nomenclature for Newcastle disease virus. Infect. Genet. Evol..

[bib0017] Dimitrov K.M. (2024). Newcastle disease in poultry. Merck Man..

[bib0018] Duan X. (2020). gga-miR-27b-3p enhances type I interferon expression and suppresses infectious bursal disease virus replication via targeting cellular suppressors of cytokine signaling 3 and 6 (SOCS3 and 6). Virus Res..

[bib0019] Duchaine T.F., Fabian M.R. (2019). Mechanistic insights into MicroRNA-mediated gene silencing. Cold Spring Harb. Perspect Biol..

[bib0020] He L., Zhang Z., Yu Q. (2020). Expression of two foreign genes by a Newcastle disease virus vector from the optimal insertion sites through a combination of the ITU and IRES-dependent Expression approaches. Front. Microbiol..

[bib0021] Hong Y. (2021). Exosomal miRNA profiling from H5N1 avian influenza virus-infected chickens. Vet. Res..

[bib0022] Hu H., Roth J.P., Yu Q. (2018). Generation of a recombinant Newcastle disease virus expressing two foreign genes for use as a multivalent vaccine and gene therapy vector. Vaccine.

[bib0023] Huang Z. (2003). Newcastle disease virus V protein is associated with viral pathogenesis and functions as an alpha interferon antagonist. J. Virol..

[bib0024] Hutcheson J.M. (2015). Delayed Newcastle disease virus replication using RNA interference to target the nucleoprotein. Biologicals.

[bib0025] Jopling C.L. (2005). Modulation of hepatitis C virus RNA abundance by a liver-specific MicroRNA. Science.

[bib0026] Kang S. (2023). Analysis of miRNA expression in the trachea of Ri chicken infected with the highly pathogenic avian influenza H5N1 virus. J. Vet. Sci..

[bib0027] Kim C. (2025). Exosomal miRNA profiling in chickens infected with highly and low pathogenic avian influenza viruses. J. Vet. Sci..

[bib0028] Langlois R.A. (2013). MicroRNA-based strategy to mitigate the risk of gain-of-function influenza studies. Nat. Biotechnol..

[bib0029] Li J. (2015). Generation of a safe and effective live viral vaccine by virus self-attenuation using species-specific artificial microRNA. J. Control Release.

[bib0030] Li J., Zheng S.J. (2020). Role of MicroRNAs in host defense against infectious bursal disease virus (IBDV) infection: a hidden front line. Viruses.

[bib0031] Mall C. (2013). Stability of miRNA in human urine supports its biomarker potential. Biomark Med..

[bib0032] Mears M.C., Bakre A. (2024). Characterizing host microRNA: virus interactions of orthoavulavirus javaense. Viruses.

[bib0033] Mohammadinasr M. (2024). Epstein-Barr virus-encoded BART9 and BART15 miRNAs are elevated in exosomes of cerebrospinal fluid from relapsing-remitting multiple sclerosis patients. Cytokine.

[bib0034] Msoffe P.L.M. (2019). Isolation and characterization of Newcastle disease virus from live bird markets in Tanzania. Avian Dis..

[bib0035] Nan F.L. (2021). Lentogenic NDV V protein inhibits IFN responses and represses cell apoptosis. Vet. Microbiol..

[bib0036] Nath Neerukonda S. (2019). Comparison of exosomes purified via ultracentrifugation (UC) and Total Exosome Isolation (TEI) reagent from the serum of Marek's disease virus (MDV)-vaccinated and tumor-bearing chickens. J. Virol Methods.

[bib0037] Neerukonda S.N. (2019). Comparison of the transcriptomes and proteomes of serum exosomes from Marek's Disease virus-vaccinated and protected and lymphoma-bearing chickens. Genes (Basel).

[bib0038] OIE (2021). Man. Diagn. Tests Vaccines Terr. Anim..

[bib0039] Park M.S. (2003). Newcastle disease virus V protein is a determinant of host range restriction. J. Virol..

[bib0040] Parris D.J., Kariithi H., Suarez D.L. (2022). Non-target RNA depletion strategy to improve sensitivity of next-generation sequencing for the detection of RNA viruses in poultry. J. Vet. Diagn. Invest..

[bib0041] Perez J.T. (2009). MicroRNA-mediated species-specific attenuation of influenza A virus. Nat. Biotechnol..

[bib0042] Qiu X. (2016). Newcastle disease virus V protein targets phosphorylated STAT1 to block IFN-I signaling. PLoS One.

[bib0043] Sakurai F. (2019). miR-27b-mediated suppression of aquaporin-11 expression in hepatocytes reduces HCV genomic RNA levels but not viral titers. Virol. J..

[bib0044] Samy A. (2025). IFITM knockout DF1 cells produce higher influenza and newcastle disease viral yields: a proof of concept for avian origin cell-based vaccine production. Vaccine.

[bib0045] Shimakami T. (2012). Stabilization of hepatitis C virus RNA by an Ago2-miR-122 complex. Proc. Natl. Acad. Sci. U. S. A..

[bib0046] Sun Y. (2019). Newcastle disease virus V protein degrades mitochondrial antiviral signaling protein to inhibit host type I interferon production via E3 ubiquitin ligase RNF5. J. Virol..

[bib0047] Teng M. (2023). Critical roles of non-coding RNAs in lifecycle and biology of Marek's disease herpesvirus. Sci. China Life Sci..

[bib0048] Thibault P.A., Wilson J.A. (2013). Targeting miRNAs to treat Hepatitis C Virus infections and liver pathology: inhibiting the virus and altering the host. Pharmacol. Res..

[bib0049] Trobaugh D.W. (2014). RNA viruses can hijack vertebrate microRNAs to suppress innate immunity. Nature.

[bib0050] Truong A.D. (2023). Small RNA sequencing and profiling of serum-derived exosomes from African swine fever virus-infected pigs. J. Anim. Sci..

[bib0051] USDA (2014). Newcastle disease response. Plan Red Book.

[bib0052] Wallis J.W. (2004). A physical map of the chicken genome. Nature.

[bib0053] Wang X., Dang R., Yang Z. (2019). The interferon antagonistic activities of the V proteins of NDV correlated with their virulence. Virus Genes.

[bib0054] Waring B.M. (2018). MicroRNA-based attenuation of influenza virus across susceptible hosts. J. Virol..

[bib0055] WOAH (2026).

[bib0056] Yang Q. (2024). Differential analysis of IBV-infected primary chicken embryonic fibroblasts and immortalized DF-1. Microbiol. Spectr..

[bib0057] Yang S. (2016). [Lentivirus delivery of the short hairpin RNA targeting NDV P gene inhibits production of the newcastle disease virus in chicken embryo fibroblasts and chicken embryos]. Bing Du Xue Bao.

[bib0058] Yao Y., Nair V. (2014). Role of virus-encoded microRNAs in Avian viral diseases. Viruses.

[bib0059] Yin M. (2023). Exosomes from hepatitis B virus-infected hepatocytes activate hepatic stellate cells and aggravate liver fibrosis through the miR-506-3p/Nur77 pathway. J. Biochem. Mol. Toxicol..

[bib0060] Yin R. (2010). Inhibition of Newcastle disease virus replication by RNA interference targeting the matrix protein gene in chicken embryo fibroblasts. J. Virol Methods.

[bib0061] Yu Z. (2023). Rapid construction of infectious clones for distinct Newcastle disease virus genotypes. Front. Vet. Sci..

[bib0062] Yue H. (2008). shRNA-triggered RNAi inhibits expression of NDV NP gene in chicken embryo fibroblast. Front. Biol. China.

[bib0063] Yue H. (2009). Short hairpin RNA targeting NP mRNA inhibiting Newcastle disease virus production and other viral structural mRNA transcription. Virus Genes.

[bib0064] Zhao W. (2013). Application of the ligation-independent cloning (LIC) method for rapid construction of a minigenome rescue system for Newcastle disease virus VG/GA strain. Plasmid.

[bib0065] Zhao W. (2015). P and M gene junction is the optimal insertion site in Newcastle disease virus vaccine vector for foreign gene expression. J. Gen. Virol..

[bib0066] Zhao X. (2016). miR-27b attenuates apoptosis induced by transmissible gastroenteritis virus (TGEV) infection via targeting runt-related transcription factor 1 (RUNX1). PeerJ.

[bib0067] Zhou D. (2018). Reticuloendotheliosis virus and avian leukosis virus subgroup J synergistically increase the accumulation of exosomal miRNAs. Retrovirology.

[bib0068] Zhuang G. (2017). A tiny RNA that packs a big punch: the critical role of a viral miR-155 ortholog in lymphomagenesis in Marek's disease. Front. Microbiol..

